# An Automated and Highly Sensitive Chemiluminescence Immunoassay for Diagnosing Mushroom Poisoning

**DOI:** 10.3389/fchem.2021.813219

**Published:** 2021-12-23

**Authors:** Jianyu Zhu, Leina Dou, Shibei Shao, Jiaqian Kou, Xuezhi Yu, Kai Wen, Zhanhui Wang, Wenbo Yu

**Affiliations:** ^1^ Beijing Key Laboratory of Detection Technology for Animal-Derived Food Safety Beijing Laboratory for Food Quality and Safety, College of Veterinary Medicine, China Agricultural University, Beijing, China; ^2^ School of Basic Medicine, Beihua University, Jilin, China

**Keywords:** mushroom poisoning, phallotoxins, automated device, high sensitivity, chemiluminescence immunoassay

## Abstract

Mushrooms containing *Amanita* peptide toxins are the major cause of mushroom poisoning, and lead to approximately 90% of deaths. Phallotoxins are the fastest toxin causing poisoning among *Amanita* peptide toxins. Thus, it is imperative to construct a highly sensitive quantification method for the rapid diagnosis of mushroom poisoning. In this study, we established a highly sensitive and automated magnetic bead (MB)-based chemiluminescence immunoassay (CLIA) for the early, rapid diagnosis of mushroom poisoning. The limits of detection (LODs) for phallotoxins were 0.010 ng/ml in human serum and 0.009 ng/ml in human urine. Recoveries ranged from 81.6 to 95.6% with a coefficient of variation <12.9%. Analysis of *Amanita phalloides* samples by the automated MB-based CLIA was in accordance with that of HPLC-MS/MS. The advantages the MB-based CLIA, high sensitivity, repeatability, and stability, were due to the use of MBs as immune carriers, chemiluminescence as a detection signal, and an integrated device to automate the whole process. Therefore, the proposed automated MB-based CLIA is a promising option for the early and rapid clinical diagnosis of mushroom poisoning.

## Introduction

Food poisoning caused by accidental ingestion of poisonous mushrooms occurs every year (Li et al., 2021). Mushroom poisoning is a major problem in Western countries, representing approximately 5.8% of total food poisonings in the U.S.A. (Bonnet and Basson, 2004; Tavassoli et al., 2019) In addition, it is a cause of high fatality rates in China. According to data from the China National Center for Food Safety Risk Assessment, mushroom poisoning accounts for 13.3% of foodborne diseases and 20.9% of fatal cases. It is worth noting that approximately 90% of deaths from mushroom poisoning are caused by peptide toxins from the genus *Amanita* (Yin et al., 2019). To date, 22 types of natural *Amanita* peptide toxins have been isolated and identified from poisonous mushrooms. According to their amino acid composition and structure, *Amanita* peptide toxins can be divided into three groups: amatoxins, phallotoxins, and virotoxins (Wieland, 1983). Among these groups, phallotoxins act the most quickly and may cause death in mice within only 2–5 h (Vetter, 1998). The metabolism of phallotoxins in urine and serum is fast; after ingestion, they are only detectable for approximately 30 h in plasma or serum and up to 72 h in urine (Rentsch, 2010). Therefore, it is necessary for patients to obtain a rapid, early diagnosis of mushroom poisoning, especially phallotoxins poisoning. To the best of our knowledge, however, there have been few studies on detection methods for mushroom toxins and even fewer on detection methods for phallotoxins.

At present, the detection of phallotoxins is primarily done using liquid chromatography–time-of-flight mass spectrometry (LC-TOF MS) (Yoshioka et al., 2014), liquid chromatography-mass spectrometry (LC-MS) (Gicquel et al., 2014), and high-performance liquid chromatography-tandem mass spectrometry (HPLC-MS/MS) (Zhang et al., 2016). However, time-consuming instrument-based methods do not meet the clinical need for a rapid diagnosis of patients with mushroom poisoning before therapy. Therefore, a straightforward and sensitive analysis of phallotoxins for the early diagnosis of mushroom poisoning is essential. Previously, the anti-phallotoxins monoclonal antibody (mAb) 3F9 was prepared as the core reagent to establish a later flow immunoassay for rapid screening of phallotoxins (Zhu et al., 2021). However, the sensitivity of later flow immunoassays is relatively low; moreover, qualitative/semiquantitative output cannot provide more information about the poisoning for subsequent therapy.

Chemiluminescence immunoassay (CLIA) is an analytical method that combines a chemiluminescence system with an immune reaction and has the advantages of high sensitivity, a wide linear range of detection, and no radioactive pollutants (Rongen et al., 1994; Pei et al., 2013) At present, CLIAs have been widely used in the fields of life science, clinical diagnosis, environmental monitoring, food safety and drug analysis (Li et al., 2017; Yang et al., 2021; Xu et al., 2020; Liu et al., 2020) In current CLIAs, 96-well plates or nitrocellulose membranes are extensively used as carriers to fix antibodies/antigens (Deng et al., 2018; Sun et al., 2020) However, their low specific surface area affects the efficiency of antigen-antibody recognition and reduces detection sensitivity. Magnetic beads (MBs), with the advantages of a large specific surface area, supermagnetism, and excellent biological compatibility, can function as reaction carriers and separation tools in the field of modern diagnostics (Liu et al., 2013; Wang et al., 2019) At the same time, Immunoassays based on MBs are easy to manipulate by an external magnetic field, enabling automated detection, which not only reduces manual operation, such as adding sample, discarding liquid and washing but also improves detection throughput and consistency (Ben Ismail et al., 2018; Lu et al., 2020) Therefore, a MB-based CLIA has the advantages of high sensitivity, a straightforward nature, and automation in the detection of food poisoning.

In this study, an automated MB-based CLIA was established for the detection of phallotoxins (phalloidin (PHD) and phallacidin (PCD)) in human serum and urine. MBs acted as carriers of coating antigens in the construction of an indirect competitive immunoassay platform. By generating chemiluminescent signals, phallotoxins can be quantified with high sensitivity. Through a pre-edited program, the whole CLIA process for phallotoxins was automatically performed. Owing to its high sensitivity, automation, and reliability, this assay could be a promising option for the rapid clinical diagnosis of mushroom poisoning.

## Materials and Methods

### Reagents and Instruments

PCD (≥90%) was purchased from Sigma-Aldrich (St. Louis, MO, United States). α-, β-, and γ-Amanitin and PHD (≥90%) were purchased from Enzo Life Sciences (Farmingdale, NY, United States). Activated biotin ester- and avidin-labeled magnetic beads (1 μm) (MBs-Avi) were purchased from Sigma–Aldrich. Bovine serum albumin (BSA) was purchased from Amresco Inc. (Solon, USA). The coating antigen (bovine serum albumin-PCD conjugates, PCD-OVA) and monoclonal antibody against phallotoxins (mAb 3F9) were produced in our laboratory, as described previously (Zhu et al., 2021). Horseradish peroxidase-labeled goat anti-mouse IgG (gtAm-HRP) was purchased from Jackson ImmunoResearch (West Grove, USA). Chemiluminescent substrate reagents were obtained from Merck Millipore (Billerica, USA). Phosphate buffered saline (PBS, pH = 7.2) and phosphate buffered saline-Tween 20 (PBST, pH = 7.2) buffers were purchased from Solaribo (Beijing, China). All other chemical reagents required for the experiments were of analytical grade and obtained from Sigma–Aldrich. Deep-well plates were purchased from Costar Inc. (St. Louis). The self-designed automatic immune analysis device was constructed in our laboratory. Human serum and urine samples were collected from different healthy volunteers. *Amanita phalloides* samples were provided by the Beijing Center for Disease Control and Prevention.

### Preparation of Immune Magnetic Beads

First, the biotinylated coating antigen was prepared. Activated biotin ester (10 μl) and PCD-OVA (100 μl) were mixed and reacted at room temperature for 4 h under dark conditions. The product was ultrafiltrated using a filtration membrane (10 kD) 3 times to obtain biotinylated PCD-OVA. Then, the diluted biotinylated PCD-OVA solution (2 μg/ml) was added to the MB-Avi solution, and the mixed solution was placed in a miniature oscillator to react for 30 min at room temperature. Based on the high affinity of biotin and avidin, the coating antigen (PCD-OVA) was attached to the surface of the MBs. Finally, the immune magnetic beads (MBs-PCD-OVA) were washed twice with PBST, redissolved to 400 μg/ml, and then stored at 4°C.

### The Automated MB-Based CLIA Process

First, the following liquids were preloaded into the 7 columns of a deep-well plate: competing reagents (20 μl of MBs-PCD-OVA and 40 μl of mAb 3F9 solution), the 1st washing liquid (150 μl of PBST), the 2nd washing liquid (150 μl of PBST), Enzyme-labeled antibody (100 μl of gtAm-HRP diluted in PBST), the 3rd washing liquid (150 μl of PBST), the 4th washing liquid (150 μl of PBST), and chemiluminescent substrate reagents (100 μl of a mixture of luminol and hydrogen peroxide). Then, 40 μl of samples with different phallotoxins concentrations were added to column No. 1. A self-designed automatic immune analysis device with a three-axis mechanical arm automatically performed the CLIA process. Based on the linear module of the three-axis mechanical arm, the operation module of the three-axis mechanical arm was constructed. The three-axis mechanical arm was equipped with AMC4030 software (Chengdu Fuyu Technology Co., Ltd.) to control the three-axis motor at the same time to control the high-precision linear motion of the three-axis mechanical arm, including point motion, round-trip motion, segmented motion and back-to-origin motion of the specified speed or distance. The motion of the mechanical arm could be controlled automatically according to the pre-edited program. The magnetic rod and stirring sleeve were manipulated by the MB control module to realize the automatic manipulation of magnetic beads, such as magnetic separation, transfer and resuspension. The process of the automated CLIA consisted of the following steps: 1) In column No. 1, the stirring sleeve was lowered into the solution and moved up and down (50 times/min) at room temperature for 15 min (stirring). Beads were then collected at the bottom of the stirring sleeve by moving the magnetic rod into the stirring sleeve for 1 min (magnetic separation). Finally, the magnetic rod and stirring sleeve were retracted, and the beads were positioned in the next column through parallel movement of the magnetic rod (transfer). 2) In column No. 2, the magnetic rod and stirring sleeve were lowered into the solution, and the beads were then released by moving the magnetic rod out of the stirring sleeve (resuspension). The stirring sleeve moved up and down rapidly (100 times/min) for 2 min (washing), followed by magnetic separation and transfer to the next column. 3) In column No. 3, the steps were the same as those in column No. 2. 4) In column No. 4, the stirring sleeve moved up and down (50 times/min) at room temperature for 15 min after resuspension (hatching), followed by magnetic separation and transfer to the next column. 5) In column No. 5, the beads were resuspended and washed, magnetically separated, and transferred to the next column. 6) In column No. 6, the steps were the same as those in column No. 5. 7) In column No. 7, the stirring sleeve moved up and down (50 times/min) at room temperature for 2 min after resuspension, and then the magnetic rod and stirring sleeve retracted. 8) Signal reading occurred. The values of relative light (RLU) were monitored using PerkinElmer (Waltham, MA, United States).

### Optimization of the Automated MB-Based CLIA

To improve the detection performance of the CLIA, several parameters that influenced the automated MB-based CLIA were optimized, including the concentration of mAb 3F9 solution (100, 50, 20, 10, and 5 ng/ml), the concentration of enzyme-labeled antibody solution (500, 250, 100, 50, and 25 ng/ml), and the pH of the reaction buffer (6.5, 7.0 7.4, 8.0, and 8.5). The influence of each condition on assay performance was evaluated by the values of RLU_max_ (the RLU value without analyte), IC_50_ (the concentration of analyte leading to 50% inhibition), and the ratio of RLU_max_/IC_50_. Under optimal conditions, standard competitive curves of phallotoxins solutions were obtained (*n* = 3) by a four-parameter logistic equation using OriginPro 9.1 (OriginLab, Northampton, MA, United States). The abscissa was the logarithm of the concentration of phallotoxins, and the ordinate was B/B_0_, where B is the value of RLU measured at a certain concentration of phallotoxins and B_0_ is the value of RLU measured in the absence of phallotoxins. The following four-parameter regression equation was used to fit the curves:

$$\text{Y}=\left(\text{A}-\text{B}\right)\text{/}\left[1+{\left(\text{X}/\text{C}\right)}^{\text{D}}\right]\;+\text{ B}$$

The sensitivity of the developed MB-based CLIA was defined as the phallotoxins concentration that achieved 50% inhibition, and the working range was determined as the IC_20_-IC_80_.

### Preparation and Analysis of Samples

Human urine can be used directly for automated MB-based CLIA analysis without sample pretreatment. For human serum, 1 ml of serum was mixed with 1 ml of methanol. The mixture was evenly mixed and centrifuged at 5,000 g at room temperature for 15 min. The clarified supernatant was blow-dried by liquid nitrogen gas and resuspended in 1 ml PBST. Then, the as-prepared serum sample was used for automated MB-based CLIA analysis. For *Amanita phalloides* samples, 0.2 g of *Amanita phalloides* powder was mixed with 1 ml water and 1 ml methanol. After 2 h of swirling, the mixture was centrifuged at 5,000 *g* at room temperature for 15 min. The supernatant was diluted with PBST and detected by the automated MB-based CLIA.

### Validation of the Automated MB-Based CLIA

UPLC-MS/MS was used to verify the accuracy and precision of the developed MB-based CLIA. The limit of detection (LOD) was calculated as the mean value of 20 blank samples (human serum or urine) plus three times the standard deviation (mean +3 SD). The accuracy of the automated MB-based CLIA was evaluated by the recovery rate. Blank samples (human serum and urine) were spiked with 0.05, 0.1, and 0.2 ng/ml phallotoxins, and the samples were pretreated according to the method described in the above section. The recovery was calculated according to the following equation: recovery = (measured concentration/spiked concentration) × 100%. The coefficient of variation (CV) was determined by analysis of the above results. The intra- and inter-assay precisions of the MB-based CLIAs were represented by the CV of three consecutive days. Furthermore, 10 *Amanita phalloides* samples were analyzed by automated MB-based CLIA and HPLC-MS/MS methods.

## Results and Discussion

### Principle of MB-Based CLIAs for Automated Detection of Phallotoxins

**FIGURE 1:**
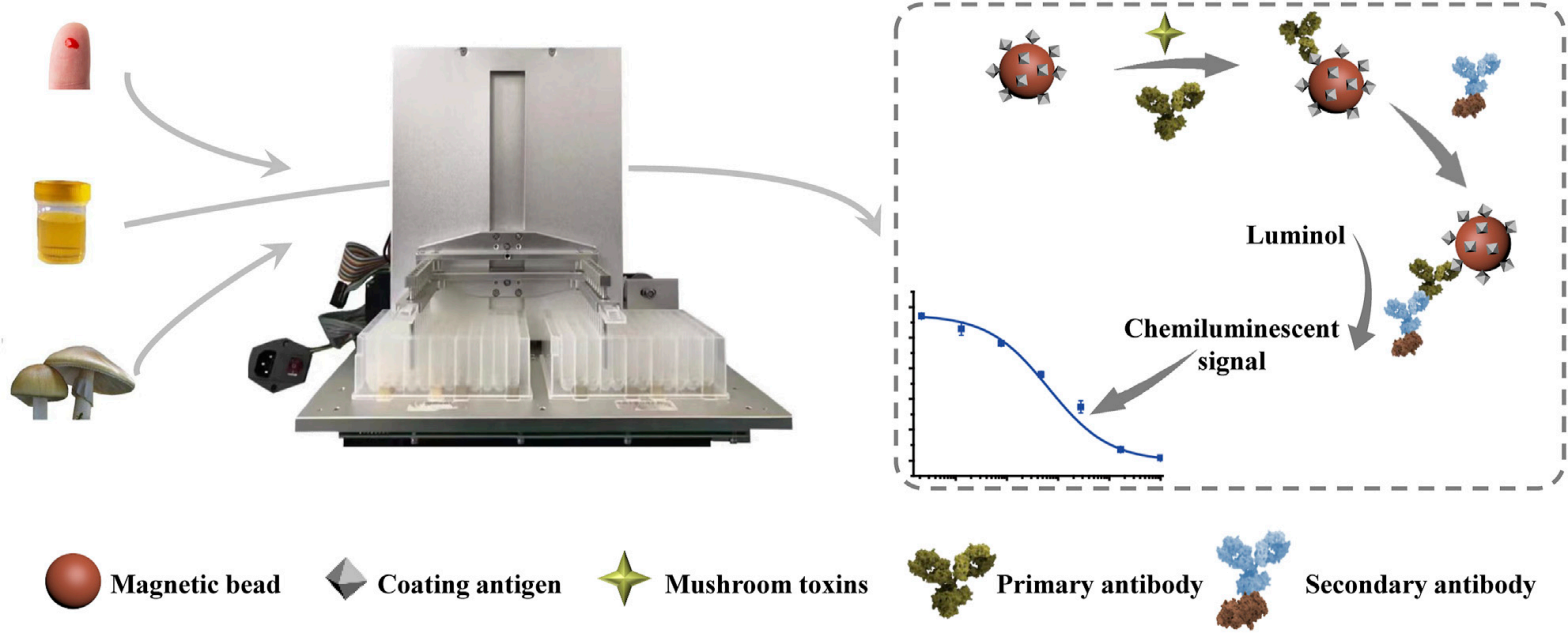
Schematic illustration of the automated MB-based CLIA for quantitative detection of phallotoxins.

In this study, the coating antigen (PCD-OVA) was coupled with MBs through the biotin-avidin system, the interaction between biotin and avidin is the strongest known noncovalent interaction. Moreover, the biotin-avidin system is a universal coupling method. In the detection of other targets, coupling between different proteins and MBs can be realized only by biotinylation of different coating proteins. The MB-coating antigen complex (MB-PCD-OVA) was used as the carrier in an indirect competitive CLIA. Immune MBs can be manipulated by an external magnetic field owing to superparamagnetism, thus realizing automated detection with high repeatability. In the automated process, the magnetic rod and stirring sleeve were driven by a self-designed automatic immune analysis device with a three-axis mechanical arm. This device accomplished stirring, magnetic separation, resuspension, washing, and hatching. The AMC4030 control software editing program was used to realize automated immune detection of phallotoxins, via the processes of immune competitive reaction, enzyme-labeled secondary antibody incubation, and chemiluminescent signal generation. The scheme for our automated MB-based CLIA is shown in Figure 1. First, the competitive immune reaction was carried out by stirring the following competing reactants: MBs-PCD-OVA, mAb 3F9, and samples of various phallotoxins concentrations. Then, the MBs were dispersed in the enzyme-labeled antibody solution for the incubation reaction after washing. Finally, the beads were transferred to a column containing chemiluminescent substrate reagent solution, and HRP catalyzed the substrate to generate chemiluminescence signals. The overall automated process for a MB-based CLIA takes only 45 min. After every step of the progress, a magnetic plate was used to magnetize the outer edge of the bottom of the deep-well plate for 5 min, and the aggregation of MBs was not seen by the naked eye. At the end of the detection process, the UV–Vis spectrogram of the liquid in each well was detected before and after the detection, and the absorption peaks were not shifted at 400–700 nm, so it was considered that the loss of MBs in the process of magnetic separation and magnetic transfer was negligible.

### Optimization of the Automated MB-Based CLIA

**FIGURE 2:**
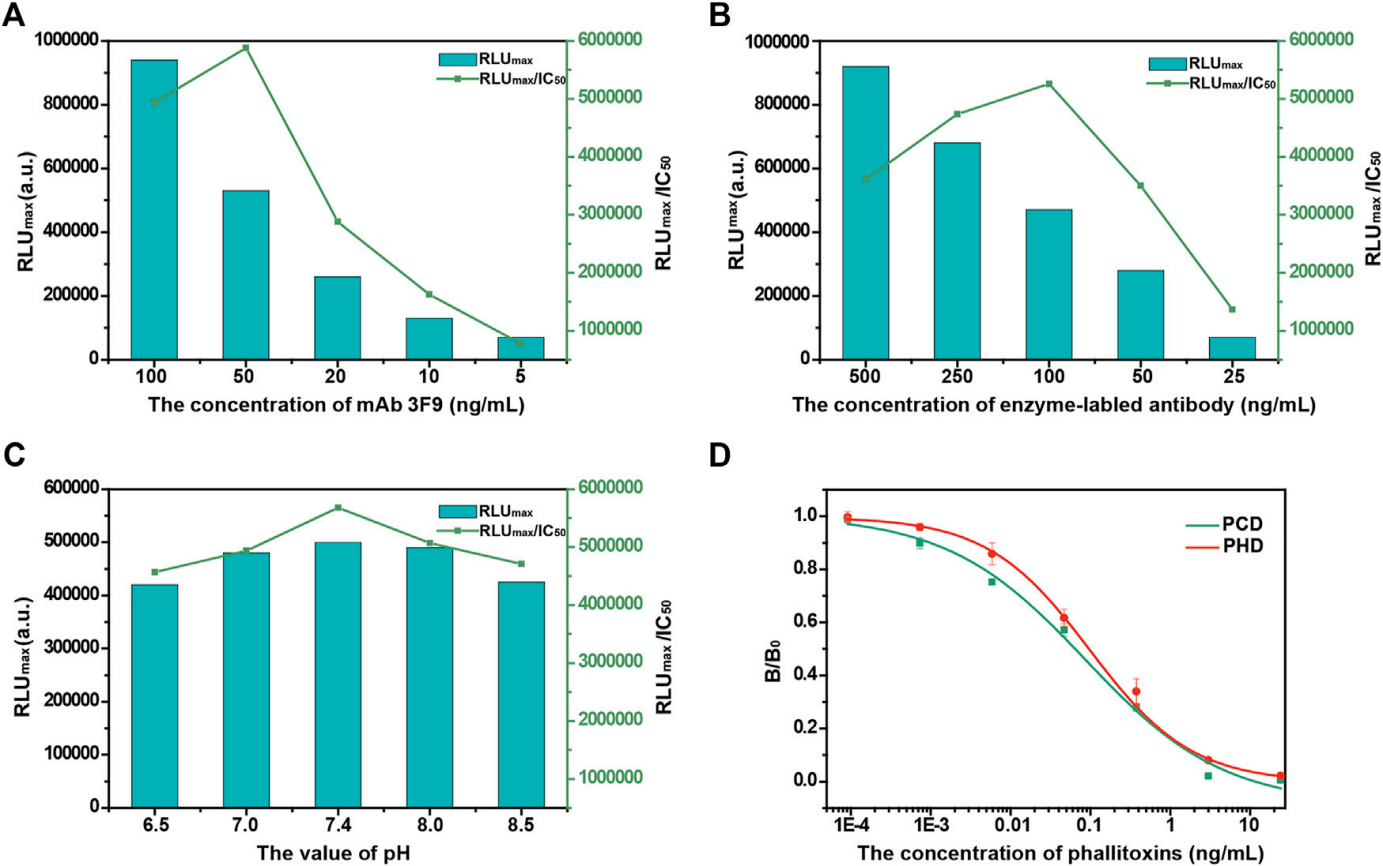
Optimization of the experimental parameters of the automated MB-based CLIA. **(A)** Concentration of mAb; **(B)** Concentration of enzyme-labeled antibody; **(C)** pH of the reaction buffer; and **(D)** Calibration curves of the automated MB-based CLIA for phallotoxins (*N* = 3).

The performance of a MB-based CLIA is related to several conditions of preparation, and a systematic optimization of pertinent parameters can improve phallotoxins detection. Here, immunoreaction parameters, including the concentration of mAb 3F9, the concentration of enzyme-labeled antibody, and the pH of buffer, were optimized. The results are shown in Figures 2A–C. RLU_max_ represents the signal intensity of the automated MB-based CLIA, and IC_50_ represents the detection sensitivity. The RLU_max_/IC_50_ ratio provides a useful parameter to assess the influence of different factors on the automated MB-based CLIA performance, with a higher RLU_max_/IC_50_ ratio (higher RLU_max_ and lower IC_50_) indicating higher signal intensity and sensitivity. The concentration of mAb 3F9 was a key factor in the development of MB-based CLIA for the detection of phallotoxins. As shown in Figure 2A, the RLU_max_ of MB-based CLIA gradually increased with the increase in the concentration of mAb 3F9, and it had the strongest intensity at 100 ng/ml. However, when the dilution concentration reached 50 ng/ml, the IC_50_ exhibited a minimum value, and at the same time, the RLU_max_/IC_50_ exhibited a maximum value. It is considered that the detection performance was better at the concentration. Based on these results, 50 ng/ml mAb 3F9 was selected as the optimal concentration. Similarly, 100 ng/ml enzyme-labeled antibody was selected to achieve better detection performance. Optimization of the pH to develop a CLIA was necessary because the pH can affect antibody activity. As shown in Figure 2C, mAb 3F9 was well tolerated to pH. In contrast, pH 7.4 was the optimal pH for MB-based CLIA.

### The Detection Performance of the Automated MB-Based CLIA

In this study, MBs were used as immune carriers to enhance the efficiency of immune recognition, chemiluminescence was applied to improve the sensitivity and working range of detection, and an integrated device was used to realize an automated detection process. Based on the above designs, a highly sensitive automated MB-based CLIA for the detection of phallotoxins was established, and the performance of this method was further investigated. The detection sensitivity of the proposed MB-based CLIA was evaluated first. Calibration curves were established using different concentrations of phallotoxins (PCD or PHD) under optimal conditions. The sensitivities (IC_50_) of the automated MB-based CLIAs were 0.087 and 0.097 ng/ml for PCD and PHD, respectively (Figure 2D). The working ranges for PCD and PHD, determined as IC_20_-IC_80_, were 0.005–1.376 and 0.013–0.751 ng/ml, respectively.

**FIGURE 3:**
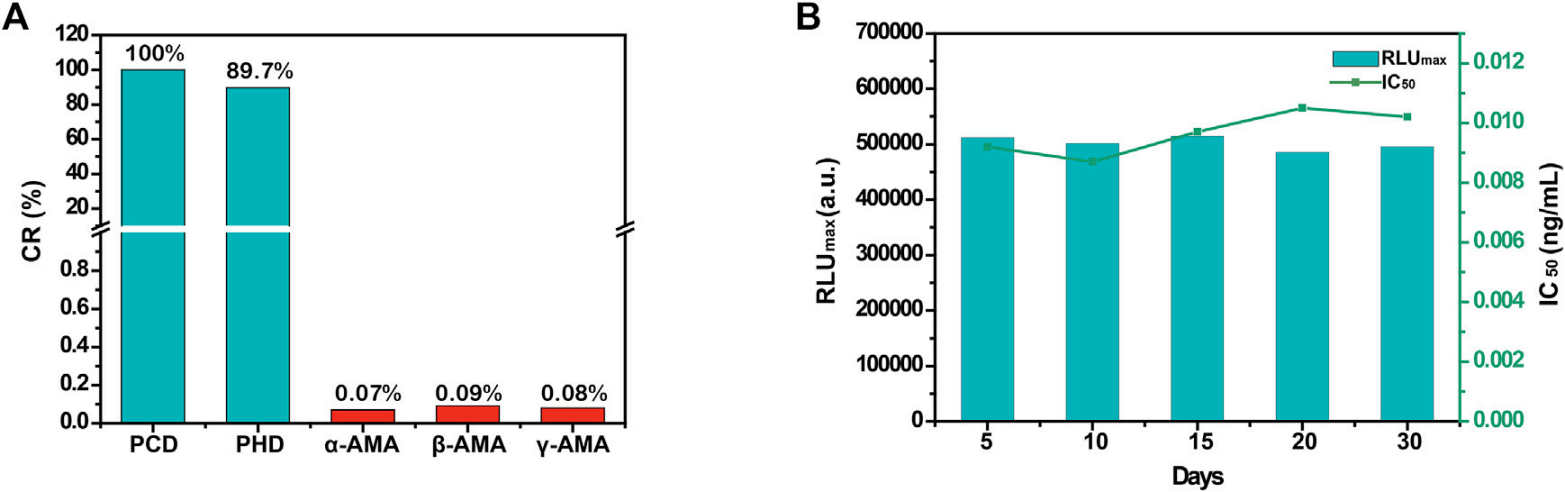
**(A)** The CRs of *Amanita peptide* toxins of automated MB-based CLIA; **(B)** The stability of the automated MB-based CLIA.

The specificity of the automated MB-based CLIA was assessed by cross-reactivity (CR) against the toxic cyclopeptides found in mushrooms of the genus *Amanita*, including α-amanitin, β-amanitin, and γ-amanitin, under the same experimental conditions. As summarized in Figure 3A, except for PCD (100%) and PHD (89.7%), the automated MB-based CLIA showed no CR (<0.1%) with α-amanitin, β-amanitin, or γ-amanitin, demonstrating the high specificity of the developed MB-based CLIA. This is important because application of the developed method depends on its repeatability and stability. Herein, repeatability was investigated by detecting PCD at different concentrations five times. The results showed that the coefficients of variation of PCD at 5 different concentrations of 0.05, 0.1, 0.2, 0.5, and 1.0 ng/ml were 5.4, 2.9, 6.8, 3.5, and 4.6%, respectively, which demonstrated superior repeatability of the automated MB-based CLIA. Furthermore, the stability of the automated MB-based CLIA was also investigated as described above. The IC_50_ value showed no obvious decrease when mAb 3F9, MBs-PCD-OVA, and enzyme-labeled antibody were stored at 4°C in PBST for 30 days (Figure 3B), indicating the excellent stability of the automated MB-based CLIA.

### Analytical Performance of the MB-Based CLIA for Phallotoxins Detection in Clinical Samples

The LODs for quantification in clinical samples were calculated as the mean value of 20 blank human serum or human urine samples plus three times the standard deviation (mean +3 SD). Each of the 20 blank samples was extracted and analyzed according to the automated MB-based CLIA procedure. The LODs for PCD and PHD were 0.010 ng/ml in human serum and 0.009 ng/ml in human urine, respectively. Notably, the detection sensitivity of the proposed MB-based CLIA is superior to that of previously reported conventional methods for phallotoxins detection (Table 1). Compared with the immunoassays established in our previous study, the LOD of the automated MB-based CLIA was one order of magnitude lower than that of an indirect competitive enzyme-linked immunosorbent assay (ic-ELISA) and time-resolved fluorescent nanosphere-based lateral flow assay (TRFN-LFA) and two orders of magnitude lower than that of a colloidal gold nanoparticle-based lateral flow assay (GNP-LFA). As a special biomolecular immobilization carrier, MBs have potentially larger surface areas, which can immobilize high-density antibodies and improve the sensitivity of detection methods. In this study, MBs were used as the carrier of CLIA. The established automated MB-based CLIA has high sensitivity and a wide working range.

**TABLE 1:** Comparison of the developed automated MB-based CLIA with other reported methods for phallotoxins detection.

Measurement methods		LOD (ng/ml)	References
Instrumental analysis	LC–TOF MS	0.22–0.37	Yoshioka et al. (2014)
	LC-MS	0.25	Gicquel et al. (2014)
	UPLC-MS/MS	0.5	Zhang et al. (2016)
Immunoassay	ic-ELISA	0.06–0.07	Zhu et al. (2021)
	GNP-LFA	3.0	Zhu et al. (2021)
	TRFN-LFA	0.1	Zhu et al. (2021)
	Automated MB-based CLIA	0.009–0.010	This work

To evaluate the accuracy of the automated MB-based CLIA, blank human serum or human urine samples confirmed by HPLC-MS/MS were combined with PCD and PHD standards at different known concentrations (0.05, 0.1, 0.2 ng/ml). Concentrations close to IC_50_ were used as the medium concentration. Then, the high and low concentrations were calculated by multiplying or dividing the medium concentration by a factor of 2, and the three spiked concentrations were all within the linear range. The average intra- and inter-assay recoveries of phallotoxins ranged from 81.6 to 95.6% with a CV less than 12.9% (Table. 2), The average intra- and inter-assay recoveries of phallotoxins ranged from 81.6 to 95.6% with a CV less than 12.9% (Table. 2), which was acceptable for the further application of the developed MB-based CLIA in real samples. This confirmed that automated MB-based CLIA was a practicable and accurate detection method and suitable for the detection of phallotoxins in human serum and urine.

**TABLE 2:** Recovery values and coefficient of variation (CV) values for phallotoxins detection in human serum and urine samples (*N* = 3)^a^.

Phallotoxins	Spiked	Working range	Human Serum			Human Urine		
	(ng/ml)		Recovery (%)	Intra-assay CV (%)	Inter-assay CV (%)	Recovery (%)	Intra-assay CV (%)	Inter-assay CV (%)
PCD	0.05	0.005–1.376	95.6	8.6	10.6	81.6	6.8	7.9
	0.1		94.1	9.5	8.5	82.2	8.0	9.4
	0.2		85.9	6.9	12.9	88.8	9.4	9.5
PHD	0.05	0.013–0.751	87.7	5.7	7.8	81.9	4.8	10.5
	0.1		89.0	4.6	4.9	94.5	9.8	6.7
	0.2		91.7	6.9	12.6	86.7	9.9	9.8

a Each concentration was in three replicates.

Then, the detection performance of the automated MB-based CLIA was further evaluated using real samples. Unfortunately, we did not obtain real serum and urine samples from patients with phallotoxins poisoning. Even so, samples from one of the deadliest mushrooms, *Amanita phalloides*, provided by the Beijing Center for Disease Control and Prevention, were detected. The quantitative results of the automated MB-based CLIA and HPLC-MS/MS were compared. As shown in Table 3, the detection results for 10 *Amanita phalloides* samples using the automated MB-based CLIA were not significantly different from those obtained using HPLC-MS/MS, which indicates that the automated MB-based CLIA could be used for the analysis of phallotoxins in poisonous mushroom samples with great effectiveness and applicability.

**TABLE 3:** Simultaneous determination of Amanita phalloides samples by automated MB-based CLIA and HPLC-MS/MS.

*Amanita phalloides* samples	Automated MB-based CLIA (g/kg)	UPLC-MS/MS (g/kg)	
	Phallotoxins	PCD	PHD
1#	2.98 ± 0.13	1.30	1.82
2#	2.24 ± 0.09	0.70	1.93
3#	3.19 ± 0.19	1.66	2.03
4#	2.06 ± 0.11	1.27	2.16
5#	1.22 ± 0.16	0.98	1.53
6#	2.30 ± 0.10	1.28	1.57
7#	3.78 ± 0.12	1.83	2.43
8#	2.56 ± 0.20	1.45	1.75
9#	1.40 ± 0.08	0.76	1.02
10#	2.66 ± 0.10	1.14	1.84

## Conclusion

In summary, MBs were used as carriers in indirect competitive CLIAs and manipulated by an external magnetic field using an integrated device. MBs had the advantages of a large specific surface area, supermagnetism, and excellent biological compatibility. Subsequently, an automated MB-based CLIA was developed and applied to detect phallotoxins in human serum and urine. The automated full immunoassay process of MB-based CLIA takes only 45 min, which is half the time compared with traditional ELISA, reduces manual operation, and improves detection throughput and sensitivity. Furthermore, the automated MB-based CLIA offers broad linear detection ranges (0.005–1.376 and 0.013–0.751 ng/ml) and low detection limits of 0.009–0.010 ng/ml. The automated MB-based CLIA has advantages of specificity, repeatability, stability, accuracy, and precision. In *Amanita phalloides* sample detection, the results from the automated MB-based CLIA fit well with results from HPLC-MS/MS. Overall, the automated MB-based CLIA established in this study has the potential to be a screening tool in the rapid clinical diagnosis of mushroom poisoning.

## Data Availability

The original contributions presented in the study are included in the article/Supplementary Material, further inquiries can be directed to the corresponding author.
